# Tacrolimus monitoring in hair samples of kidney transplant recipients

**DOI:** 10.3389/fmed.2023.1307505

**Published:** 2023-12-04

**Authors:** Alexander Born, Federica Bocchi, Christian Kuhn, Ursula Amstutz, Markus R. Baumgartner, Daniel Sidler

**Affiliations:** ^1^Department of Nephrology and Hypertension, Inselspital, Bern University Hospital, University of Bern, Bern, Switzerland; ^2^Department of Clinical Chemistry, Inselspital, Bern University Hospital, University of Bern, Bern, Switzerland; ^3^Center for Forensic Hair Analytics, University of Zurich, Zurich, Switzerland

**Keywords:** kidney transplantation, tacrolimus, C_0_ level, mass-spectrometry, hair, trough levels

## Abstract

**Background:**

Calcineurin inhibitors, including tacrolimus, remain a cornerstone of immunosuppressive therapy after kidney transplantation. However, the therapeutic window is narrow, and nephrotoxic side effects occur with overdose, while the risk of alloimmunization and graft rejection increases with underdose. Liquid chromatography-tandem mass spectrometry (LC-MS/MS) allows quantification of tacrolimus in biological samples from patients. This study investigates the feasibility of quantifying tacrolimus in scalp hair from kidney transplant (KT) recipients and correlates hair tacrolimus concentrations with tacrolimus dosage and blood trough levels. The aim was to provide proof-of-principle for hair tacrolimus drug monitoring in KT recipients.

**Method:**

Single-center prospective study between September 9, 2021 and December 4, 2021, including KT recipients under tacrolimus. Minors, patients with active skin or hair diseases, and patients with scalp hair shorter than 4 cm were excluded from participation. Scalp hair was collected from the posterior vertex of patients, cut into segments, and analyzed for tacrolimus by LC-MS/MS. Patients filled out a questionnaire on hair treatments and washing habits. In parallel, tacrolimus trough levels were measured in whole blood and correlated with hair tacrolimus concentrations.

**Results:**

In total, 39 consenting KT recipients were included, and hair samples were collected at 53 visits. Tacrolimus was detected in 98% of hair samples from patients exposed to the drug. Tacrolimus hair levels and whole blood trough levels were correlated with a beta coefficient of 0.42 (95% CI: −0.22–1.1, *p* = n.s.). Age and dark hair affected hair tacrolimus measurements, while different tacrolimus formulations (immediate release vs. extended release), hair washes, and permanent coloring did not. Longitudinal measurements in a subgroup of patients indicate that long-term measurement of hair tacrolimus levels is feasible.

**Conclusion:**

Measuring tacrolimus in hair is a potentially reliable method to monitor drug exposure in KT patients. Rapid wash-in effects and consistent concentrations over time indicate that tacrolimus is incorporated into the hair matrix, allowing temporal resolution in the analysis of recent exposure and exposure history. This method provides a simple and low-risk alternative to regular blood sampling, sparing patients from frequent hospital visits through the self-collection of hair samples.

## Introduction

Kidney transplantation (KT) is an effective treatment for advanced and end-stage kidney disease (1, 2). Although short-term outcomes have improved substantially over the last decades, long-term results are still unsatisfactory (3). The primary causes of allograft failure remain chronic antibody-mediated rejection due to relative under-immunosuppression and calcineurin inhibitor (CNI) toxicity. The latter reflects a common nephrotoxic side effect of CNI, namely, cyclosporine A (CsA) and tacrolimus (Tac) (4–6). While these agents represent a cornerstone in the treatment of solid transplant recipients, they have a narrow therapeutic range and pose a substantial toxicity risk if overdosed. In particular, the nephrotoxic effects of these drugs may lead to progressive allograft disease and premature graft failure (7). Furthermore, CNI elicits extra-renal side effects, including progressive cardio-vascular disease (8), vulnerability against infections, and risk for cancer (9), all of which contribute to increased morbidity and mortality in KT recipients (10).

In the past decades, much effort has been made to measure CNI exposure and to adjust treatment doses to pre-specified target CNI blood levels for individual patients (11, 12). Indeed, tailoring immunosuppressive therapy to each individual KT recipient is a good example of precision- and patient-centered medicine (13). Unfortunately, these efforts have not yet led to substantial breakthroughs since CNI blood levels only poorly correlate with toxicity and we cannot predict whether CNI toxicity will progress or not (14).

Drugs and metabolites can be analyzed by liquid chromatography-tandem mass spectrometry (LC-MS/MS) from biological matrices, including blood (15), hair (16), and nails (17). In forensic toxicology, retrospective quantification of chemicals in hair samples has gained widespread acceptance. Chemicals such as cocaine (18), ethyl glucuronide (19), and delta9-THC (20) are quantified to confirm abstinence in patients who are recovering from addiction (21, 22). Furthermore, long-term medication monitoring in hair is feasible, accurate, and predictive in specific clinical settings. For instance, tenofovir concentrations measured in hair samples can be readily used to monitor treatment adherence in HIV patients (23). However, although LC-MS/MS analysis of substances in hair is specific and sensitive, certain factors, such as hair products, hair washing routines, hair color, and artificial coloring, are known to significantly affect the results of hair analysis (20, 24, 25).

The aim of this trial was to quantify tacrolimus in the scalp hair of KT recipients and correlate concentrations with tacrolimus dosing and blood C_0_ levels.

## Methods

### Study design and population

This study evaluates a subgroup of the Bernese transplant cohort. KT recipients on maintenance therapy with tacrolimus (Prograf^®^, Advagraf^®^, or Envarsus^®^) were screened and enrolled in the study during routine outpatient follow-up at the Nephrology Department of the University Hospital Insel in Bern between September 9, 2021 and December 4, 2021. Minors, patients without at least 4 cm long hair in their vertex, and patients with active skin or hair diseases were ineligible to participate. The study was approved by the Local Ethics Committee (2020-00953). All patients provided oral and written consent.

### Clinical and laboratory parameters

Baseline characteristics and treatments were extracted from the electronic patient documentation. Information on hair color, care, and utilized hair treatment products was collected with a questionnaire. Tacrolimus concentration was determined 12 h after the last dose of immediate-release tacrolimus (Prograf^®^) and 24 h after the last dose of extended-release tacrolimus (Advagraf^®^ or Envarsus^®^). The daily tacrolimus dose was recorded as a cumulative dose in mg per day. Serum creatinine was measured from plasma samples; eGFR was estimated according to the CKD-EPI equation (26) and expressed in mL/min/1.73 m^2^.

### Hair sampling and processing

Patients were allowed to provide hair specimens at multiple study visits. Specifically, a strand of hair with a diameter of 2–4 mm was cut at the base from the posterior scalp of participants. The end of the hair tuft adjacent to the scalp was marked. The bottom proximal 2 cm segment (S1) and the adjacent 2 cm segment (S2) of the specimens were segmented and used for further analysis. Hair specimens were cleaned, chopped into snippets, ground into a powder, and then utilized for mass spectrometry analysis. First, hair samples were cut into segments of exact length, and each segment was decontaminated with the following standard protocol for forensic hair analysis. The hair was washed once with 5 mL of deionized water and twice with 5 mL of acetone for 3 min each. After drying at room temperature, hair segments were chopped into snippets using scissors. For extraction, between 5 and 25 mg of snippets were exactly weighed into an Eppendorf vial, and the snippets were pulverized for 15 min at 30 Hz. Then, 100 μL of IS solution and 1,400 μL of methanol were added, and the samples were sonicated for 2 h at 40°C. After centrifugation for 10 min at 9,000 g, the supernatant clear solution was transferred to a vial for evaporation under a stream of nitrogen at 40°C. For injection into the LC-MS/MS system, the residue was reconstituted in 30 μL of methanol and 70 μL of 5 mM ammonium formate (pH 3) with 10% (v/v) of methanol.

### Preparation of working solutions

Spiking solutions for calibrators and quality control were prepared in methanol to obtain concentrations comparable to those found in hair. As an internal standard (IS), a solution was prepared in methanol containing 13CD_4_-tacrolimus at a concentration of 800 pg/mg at a sample weight of 1 mg.

### LC-MS/MS parameters

The LC–MS-MS system consisted of a Shimadzu Prominence high-performance liquid chromatography system (Shimadzu, Duisburg, Germany) and a QTrap 6500 mass spectrometer (Sciex, Darmstadt, Germany) using electrospray ionization (ESI) operating in positive mode. Separation was achieved using a Kinetex^®^ F5 column (100 × 2.1 mm, 100 Å, 2.6 μm, Phenomenex) coupled with SecurityGuard™ ULTRA Cartridges ultra-high performance liquid chromatography (UHPLC) F5 (2.1 mm ID). A mobile phase A [water containing ammonium formate (1 mM) and formic acid (0.1%)] and a mobile phase B [acetonitrile containing ammonium formate (1 mM) and formic acid (1 mM)] were used. A post-column spray of methanol was applied with a flow rate of 0.04 mL/min to support the ionization process. The flow rate was set at 0.6 mL/min, and the gradient was programmed as follows: 0.01–1.5 min, 10% eluent B; 1.5–9 min increasing to 95% eluent B; 9–11 min, 95% eluent B; 11–11.1 min decreasing to 10% eluent B; and 11.1–12 min starting conditions (10% eluent B). The column oven was set at 40°C. The dead time (t0) was about 0.3 min (0.19-mL void volume of the column). The autosampler was operated at 15°C, and the autosampler needle was rinsed before and after aspiration of the sample using methanol. The mass spectrometry (MS) instrument was operated in the “Scheduled MRM™ Algorithm Pro” mode. Quantification was achieved by calculating the mean concentration of both transitions. MRM transitions and retention times of tacrolimus and 13CD_4_-tacrolimus (IS) are given in Supplementary Table 1. The following identification criteria were used: (1) the retention time (RT) between the analyte and the IS and (2) deviations ≤ 20% for the relative area ratios of the three transitions (MRM 1 to MRM 2 and MRM1 to MRM3, respectively).

### Calibration curve and method validation

Three calibration concentrations (C1–C3) and a blind hair sample were prepared to establish the linearity of the calibration. Approximately 20 mg of tacrolimus-free hair was analyzed without or spiked at concentrations C1–C3. The regression was calculated using a linear model (Supplementary Table 2). The method was partially validated for the selected parameters, namely, selectivity, the lower limit of detection (LLOD), the lower limit of quantification (LLOQ), and linearity. Tacrolimus hair concentration (hC_0_) was measured in picograms per sample (pg/sample) and normalized to the input weight, resulting in a hair tacrolimus concentration (pg/mg).

### Longitudinal sampling

Drugs and metabolites are transported to the hair follicle via the bloodstream and permanently incorporated into the matrix. Over time and along with hair growth, the matrix moves away from the follicle and remains relatively inert in terms of component incorporation and washout. To test this notion, we analyzed hC_0_ levels in the S1 segment of visit 1 (representing recent tacrolimus exposure) and the S2 segment of visit 2 (representing tacrolimus exposure 2–4 months ago). Furthermore, we compared hC_0_ in S1 and S2 segments in two patients, one with recent tacrolimus withdrawal due to belatacept-conversion and one with recent tacrolimus exposure for *de novo* KT.

### Statistical analysis

Results were reported as the number of participants (percentage) for categorical data and the median (interquartile range) for continuous data. To assess correlations between hC_0_ and drug exposure, we employed a linear regression model with hC_0_ as the dependent variable and daily dose (mg/day) as the independent parameter without (a crude model) or with potentially interfering patient-related (partial model) or cosmetic treatment-related cofactors (full model). Data were presented using histograms and xy-plots. The Pearson correlation coefficient between hC_0_ and bC_0_ (blood tacrolimus concentration) and the daily tacrolimus dose were calculated. A two-tailed *p*-value below 0.05 was considered statistically significant. Statistical analyses were performed using R (version 4.0.3) and R Studio (version 1.3.1093).

## Results

### Overall characteristics of participants and hair samples

The study cohort includes 39 KT recipients of the Bernese Transplant project. Baseline characteristics are given in Table 1. 62% of patients were female, had a median age of 53.1 years (IQR: 42.0–63.4), and had a median transplant history of 2.8 years (IQR: 0.4–6.9) at study inclusion. In total, 19% of patients suffered from glomerulonephritis as an underlying disease. A total of 24 patients (61%) were under immediate-release tacrolimus (Prograf^®^) and the remainder were under extended-release tacrolimus (Advagraf^®^, Envarsus^®^). In total, 74% were under low-dose prednisolone, and 83% were under antimetabolites (azathioprine, mycophenolate mofetil, or acetate). The average daily tacrolimus dose was 4.5 mg (IQR: 3–6) and the average trough (C_0_) level was 6.2 ng/mL (IQR: 4.6–8.0). A total of 37 patients had been taking tacrolimus for at least 6 months prior to study entry; one patient started within 2 months before entry (recent KT); and one patient was switched to belatacept between two samplings. Characteristics of hair color and treatment are given in Table 2.

**Table 1:** Baseline characteristics of study population.

	**Prograf *n* = 24**	**Advagraf *n* = 11**	**Envarsus *n* = 4**	**Overall *n* = 39**	***p*-value**
Sex (female)	15 (62%)	7 (64%)	2 (50%)	24 (62%)	>0.9
Age (years)	53.5 (42.5, 60.0)	50.2 (41.4, 60.8)	57.3 (44.0, 71.5)	53.1 (42.0, 63.4)	0.8
eGFR (mL/min/1.73 m^2^)	49 (32, 62)	45 (40, 52)	63 (39, 73)	47 (32, 63)	0.9
Tac dose (mg/day)	5.0 (3.9, 6.0)	4.0 (2.5, 4.8)	4.5 (3.2, 5.0)	4.5 (3.0, 6.0)	0.2
KT history (years)	3.8 (0.4, 9.9)	2.4 (0.4, 3.5)	1.4 (0.7, 2.0)	2.8 (0.4, 6.9)	0.2
Tac C_0_ (ng/mL)	7.1 (5.8, 9.2)	5.1 (4.1, 6.4)	7.0 (5.6, 8.7)	6.2 (4.6, 8.0)	0.093

Values are given as frequencies (percentages) or median values (interquartile range).

KT, kidney transplant; eGFR, estimated glomerular filtration rate; Tac C_0_, tacrolimus trough levels.

**Table 2:** Characteristics of the acquired S1 samples comparing different tacrolimus formulations.

**Characteristics**	**Prograf *N* = 24**	**Advagraf *N* = 11**	**Envarsus *N* = 4**	**Overall *n* = 39**	***p*-value**
Hair color					0.2
Blond	9 (38%)	6 (60%)	0 (0%)	15 (39%)	
Brown	8 (33%)	3 (30%)	1 (25%)	12 (32%)	
Black	3 (12%)	1 (10%)	1 (25%)	5 (13%)	
Gray	4 (17%)	0 (0%)	2 (50%)	6 (16%)	
Artificial coloring	6 (26%)	3 (30%)	0 (0%)	9 (24%)	0.7
Permanent structural alteration	1 (4.3%)	1 (10%)	1 (25%)	3 (8.1%)	0.2
Bleached	1 (4.2%)	0 (0%)	0 (0%)	1 (2.6%)	>0.9
Washes per week	3.0 (2.0, 7.0)	3.0 (2.1, 5.5)	3.0 (2.5, 3.5)	3.0 (2.0, 7.0)	>0.9

### Tacrolimus trough (C_0_) levels in hair and blood samples

Overall, 53 samples were collected. Eight patients participated twice, and three patients participated three times. Of the hair specimens collected, the median weight of S1 segments was 14 mg (IQR: 10–20) and S2 segments was 13 mg (IQR: 9–20). Overall, tacrolimus was detectable in 52/53 samples (98%) with a median concentration of 7.0 pg/mg (IQR: 3.5–11.0) in S1 and in 30/32 samples (93.8%) with a median concentration of 4.0 pg/mg (IQR: 2.0–6.5) in S2 (Supplementary Table 3; Supplementary Figure 1).

### Correlation of tacrolimus trough levels in hair (hC_0_), blood (bC_0_), and daily dose

**Figure 1:**
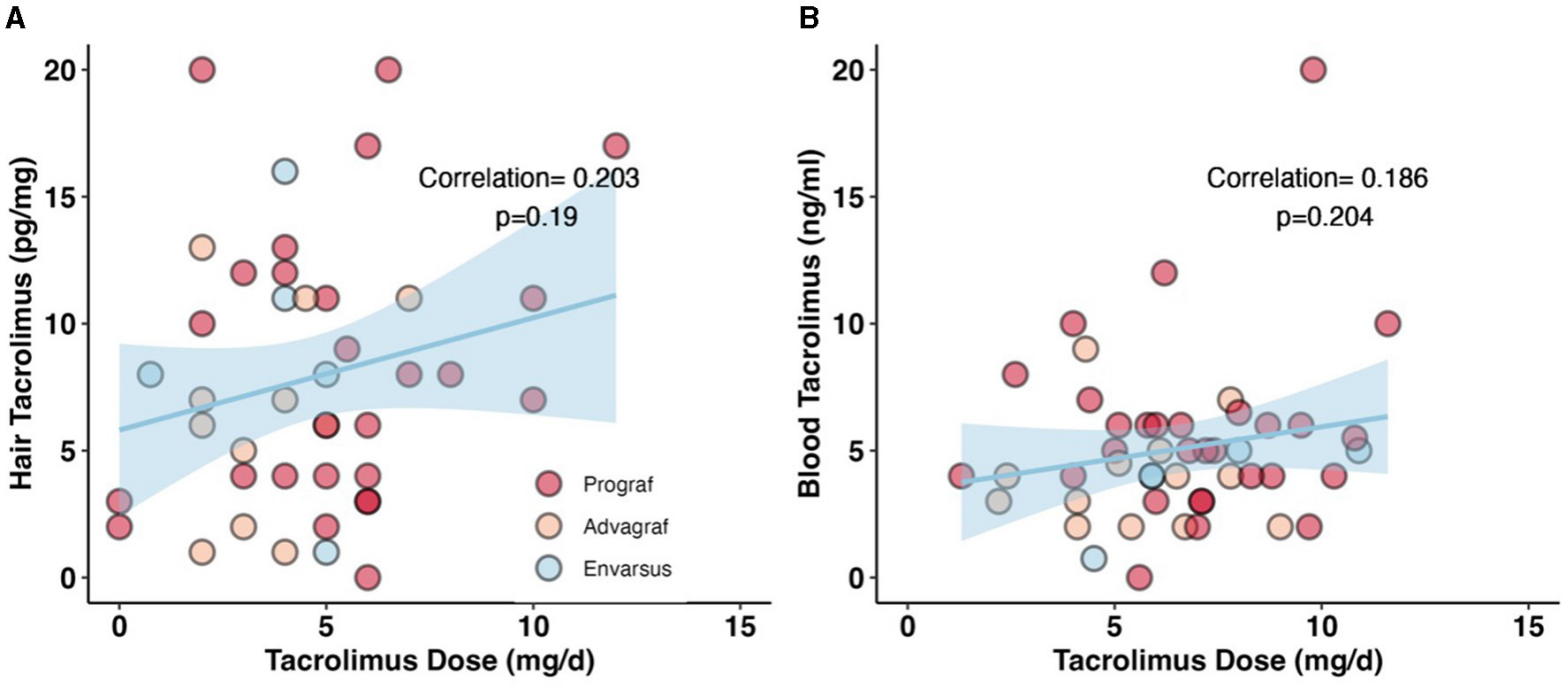
Correlation between daily dose of tacrolimus and measured drug levels in hair and blood. **(A)** Correlation between hC_0_ and daily tacrolimus dose. Pearsons correlation coefficient is 0.203, *p* = 0.19. A non-significant positive correlation between the daily tacrolimus dose and the measured hC_0_ levels. **(B)** Non-significant correlation between bC_0_ and daily tacrolimus dose. Pearsons correlation coefficient is 0.186, *p* = 0.204. In this sample we could show a stronger correlation between hC_0_ and daily dose than with bC_0_.

hC_0_ was positively correlated with daily dose (beta coefficient 0.42 per mg tacrolimus, 95% CI: −0.22 to 1.1, *p* = 0.2) in the crude model and remained positively correlated with a similar coefficient after correction for age, sex, and drug formulation (partial model, beta 0.45, 95% CI: −0.14 to 1.0, *p* = 0.13). After additional correction for hair washes, permanent structural alteration, and dark hair color, the results remain unchanged (full model, beta 0.36, 95% CI: −0.27 to 1.0, *p* = 0.3). Tacrolimus formulation had no impact on the interaction between hC_0_ and daily exposure (Table 3). Patient age negatively and dark hair positively influenced hC_0_ values. hC_0_ correlated with daily dose with a Pearson correlation coefficient of 0.203, while the correlation of bC_0_ and dose was 0.186 (Figure 1).

**Table 3:** Linear regression models for hC_0_ level in segment 1 with independent parameters.

	**Crude model**			**Partial model**			**Full model**		
	**Beta**	**95% CI**	***p*-value**	**Beta**	**95% CI**	***p*-value**	**Beta**	**95% CI**	***p*-value**
Tacrolimus dose (mg/day)	0.42	−0.22, 1.1	0.2	0.45	−0.13, 1.0	0.13	0.35	−0.28, 0.98	0.3
Tacrolimus formulation (extended release)				0.03	−3.0, 3.0	>0.9	−1.4	−4.7, 1.8	0.4
Sex (female)				−1.3	−4.1, 1.6	0.4	−1.2	−4.8, 2.4	0.5
Age (per year)				−0.2	−0.29, −0.10	**< 0.001**	−0.09	−0.22, 0.03	0.15
Washes (per week)							0.25	−0.43, 0.93	0.5
Hair color (brown/black)							3	−0.48, 6.6	0.088
Artificial coloring (yes)							2.2	−3.9, 8.2	0.5

Crude model: hC_0_ in relation to daily tacrolimus dose. Partial model: hC_0_ in relation to daily tacrolimus dose, tacrolimus formulation (extended vs. immediate release), sex, and patient age. Full model: Partial model and the parameters, namely, reported washes per week, hair color (dark hair vs. fair hair), and permanent treatment (yes). Beta coefficient, 95% confidence intervals (CI), and values are given. hC_0_, hair tacrolimus level. Bold values are significant *p*-values (<0.05).

### Longitudinal hair tacrolimus concentrations

**Figure 2:**
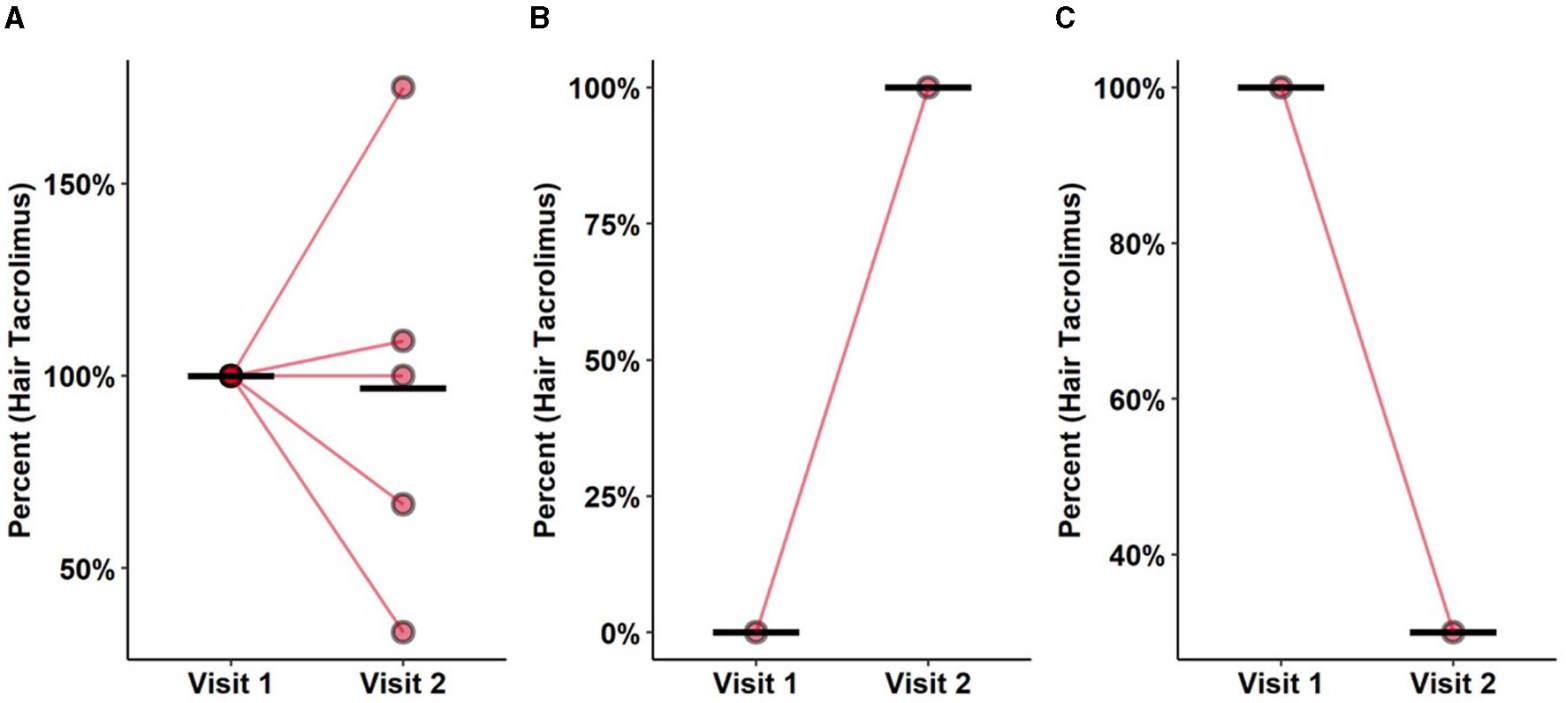
Stability over time, washin and washout. **(A)** Washout effect of hC_0_ in patients with constant (±20%) bC_0_ concentrations. S1 segment of the first visit was compared to S2 of the second visit, representing roughly the same time frame. Each segments represents 2 months of tacrolimus ingestion. S1 segment was cut of just above the skin therefore roughly representing the 2 months prior to analysis. Mean concentration was constant among both samples. Rather wide distribution of values between the samples indicates the presence of cofounders impacting stability of tacrolimus in hair. **(B)** Positive quantification in one patient *de novo* taking tacrolimus, comparing S1 segments of both visits showing a washin effect. **(C)** Washout effect after changing from tacrolimus to belatacept between two visits. Comparing S1 segments of both visits. Prompt washin and washout effects suggesting dose related incorporation in the hair. With measurable washin and out effects over a short period of time the possibility of temporal resolution in hair measurements is likely.

For five subjects in the study cohort with no change in tacrolimus medication, a hair sample was collected at visits 1 and 2, ~2 months apart. These hair samples were analyzed in segments. Assuming a hair growth rate of 1 cm/month, the proximal segment S1 of the visit 1 sample and the distal segment S2 of the later visit 2 sample represent approximately the same time period. The corresponding hC_0_ values are shown in Figure 2A.

One patient with recent KT was started on tacrolimus at the time of the first visit. Both proximal S1 segments of visits 1 and 2 were compared, and tacrolimus was detected only at the second visit (Figure 2B). Conversely, one patient changed immunosuppressive treatment from tacrolimus to belatacept between the two visits. Comparison of hC_0_ in the S1 segment at the two visits showed a decrease of 40% from the first measurement (Figure 2C).

## Discussion

To the best of our knowledge, this is the first study assessing tacrolimus hair concentration in KT recipients and correlating results with patient-related and hair treatment-related cofactors. In the vast majority of patients, tacrolimus was detectable in the hair specimen collected from the vertex. The correlation of matrix levels (hair and blood) with daily tacrolimus exposure was rather low, yet higher in hair samples compared to blood. A correlation between hC_0_ and bC_0_ was not significant. The continuous deposition of tacrolimus in growing hair is supported by the analysis of patients with recent tacrolimus withdrawal and exposure. Together, these findings strongly support the assumption that tacrolimus is incorporated into the hair matrix via the bloodstream and thereafter remains detectable weeks to months after exposure, with only limited washout effects from hair washing and hair product applications. Patient age significantly influenced results, while results were reliable and comparable among all tacrolimus formulations (Prograf^®^, Advagraf^®^, and Envarsus^®^).

The main differences were found between patients with different hair colors. Thus, there is a higher hC_0_ in patients with darker hair (brown and black). Differing levels of metabolites depending on hair pigmentation are described in hair analyses of a variety of different drugs (27, 28). Gray hair naturally correlates with increased age; we interpret the lower hC_0_ levels in older patients as a consequence of a higher fraction of gray hair.

Prednisolone has been described to induce CYP3A and/or P-glycoprotein, therefore increasing the needed tacrolimus dose to reach the target bC_0_, especially after transplantation (29). In our population, the majority of patients were on low-dose prednisolone. Prednisolone maintenance therapy had no impact on Tacrolimus concentrations in hair.

This study highlights new opportunities for therapeutic drug monitoring. First, our approach enables therapeutic drug monitoring from biological samples, independent of blood collection. Hair specimens are easily accessible and may even be collected by patients themselves or their relatives. Furthermore, sampling is independent of healthcare facilities, does not require pre-analytical processing (centrifugation and cooling), and poses a negligible risk of transmission of infectious diseases. Since hC_0_ concentrations appear to be relatively stable during the course of hair growth, this method could even be used to quantify tacrolimus exposure for weeks to months in the past.

Our study has several limitations. First, the cohort is small and comprises mainly single-time point evaluations. Second, patient- and hair treatment-associated confounders were associated with hC_0_ levels. The sample size was too small and the sampling procedures too limited to test whether these confounders remain stable over time on a patient level and whether natural or hair treatment-related changes (graying of hair in aging patients, new hair products, or permanent coloring) affect longitudinal hC_0_ values. Likely, further confounders have not been captured in detail, notably ethnic differences, given the predominance of Caucasian patients in this study. Although there is a wide distribution of pigmentation in hair, it is controversial if ethnicity affects hair analysis (28). Finally, tacrolimus and chronic kidney disease are known causes of alopecia (30, 31). However, not all patients were eligible for participation; notably, bald patients (predominantly elderly men) had to be excluded.

## Conclusion

Tacrolimus detection in patient hair offers a reliable method to quantify drug exposure, including longitudinal measurements. Further studies are needed to determine therapeutic target levels for tacrolimus hair measurements and to quantify the effects of age, hair color, and different hair treatments on hC_0_ and washout effects.

## Data availability statement

The raw data supporting the conclusions of this article will be made available by the authors, without undue reservation.

## Ethics statement

The studies involving humans were approved by Kantonale Ethikkomission Bern, Switzerland Nr. 2020-00953. The studies were conducted in accordance with the local legislation and institutional requirements. The participants provided their written informed consent to participate in this study. Written informed consent was obtained from the individual(s) for the publication of any potentially identifiable images or data included in this article.

## Author contributions

AB: Data curation, Formal analysis, Investigation, Software, Visualization, Writing—original draft. FB: Writing—review & editing. CK: Writing—review & editing. UA: Project administration, Writing—review & editing. MB: Conceptualization, Formal analysis, Methodology, Resources, Writing—review & editing. DS: Conceptualization, Data curation, Funding acquisition, Methodology, Software, Supervision, Validation, Visualization, Writing—original draft.
