# Supplementary material for: Tacrolimus monitoring in hair samples of kidney transplant recipients
Source: Front Med (Lausanne). 2023 Dec 4;10:1307505. doi: 10.3389/fmed.2023.1307505 (PMC10726046; doi:10.3389/fmed.2023.1307505)

**Supplementary Tables**

*Supplementary table 1:* LC-MS/MS Parameters

|  | Transition | Precursor ion  (m/z) | Product ion  (m/z) | Relative ion intensity | RT (min) |
| --- | --- | --- | --- | --- | --- |
| Tacrolimus | MRM1 | 821.5 | 768.30 | 1.00 | 6.57 |
|  | MRM2 |  | 786.40 | 0.48 | 6.57 |
|  | MRM3 |  | 576.20 | 0.25 | 6.57 |
| 13CD_4_-tacrolimus | MRM1 IS | 826.8 | 773.50 |  | 6.56 |

*Supplementary table 2:* Calibration

|  | Concentration (pg/mg), calculated for a sample weight of 1 mg hair | | | |
| --- | --- | --- | --- | --- |
| Calibrator | Blind hair | C1 | C2 | C3 |
|  | 0.0 | 100 | 500 | 1000 |

**Supplementary Figures**

Supplementary Figure 1: a) Distribution of hC_0_ measurements. Tacrolimus was found in all but one sample. b) Distribution of bC_0_ measurements.


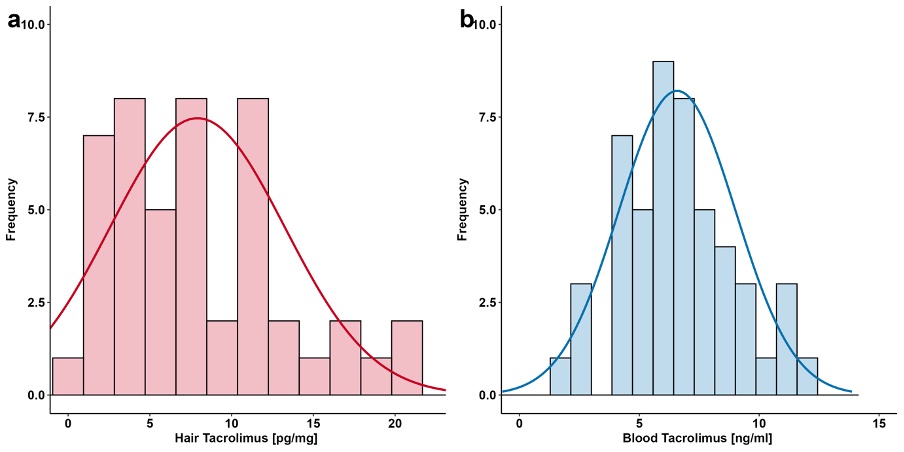

Supplement: Supplementary file 1 [file Data_Sheet_1.docx]
